
JOURNAL INFORMATION
==============================
NLM Title Abbreviation: ScientificWorldJournal
Journal ID: ScientificWorldJournal
NLM Journal ID: 101131163
Journal ID: TSWJ

The Scientific World Journal

ISSN: 2356-6140
EISSN: 1537-744X
Publisher: Wiley

ARTICLE INFORMATION
==============================
PMCID: PMC6084531
PMID: 30147489
DOI: 10.1100/tsw.2001.146
Article ID: 826142
Article version: 1
Subjects: Short Report

Pretreatment of the Cells With Brefeldin A and Cycloheximide Differently Regulates Fas and TNF Stimulated Apoptosis

Neznanov Nickolay *
Gudkov Andrei
Department of Molecular Genetics, MC669, University of Illinois at Chicago, 900 South Ashland Ave., Chicago, Illinois 60607, USA
*Nickolay Neznanov: neznanov@uic.edu
Publication date: 2001
Electronic publication date: 2001 Jun 8
Volume: 1
First page: 40
Last page: 40
Copyright: Copyright © 2001 Nickolay Neznanov and Andrei Gudkov.
Copyright year: 2001
License: This is an open access article distributed under the Creative Commons Attribution License, which permits unrestricted use, distribution, and reproduction in any medium, provided the original work is properly cited.
License URL: https://creativecommons.org/licenses/by/3.0/

==============================
Miami *Nature Biotechnology* Short Reports
*TheScientificWorld* (2001) 1(S3), 40SR
ISSN 1532-2246; DOI 10.1100/tsw.2001.146

### PRETREATMENT OF THE CELLS WITH BREFELDIN A AND CYCLOHEXIMIDE DIFFERENTLY REGULATES FAS AND TNF STIMULATED APOPTOSIS

Nickolay Neznanov*, Andrei Gudkov

Department of Molecular Genetics, MC669, University of Illinois at Chicago, 900 South Ashland Ave., Chicago, Illinois 60607
* Neznanov@uic.edu

**INTRODUCTION.** The activation of Fas and TNF receptor by ligands stimulates fast development of apoptosis in sensitive cells. The activation of these two-death domain-containing receptors leads to the stimulation of very similar, but not identical pro-apoptotic pathways. The treatment with Fas ligand alone is enough to stimulate apoptosis, but TNF-stimulated apoptosis requires the co-treatment with TNF and suppressors of translation or transcription. Here we are reporting a new difference between activation of apoptosis with Fas ligand and TNF. The pretreatment of the cells for 2-3 hours with cycloheximide or brefeldin A makes the cells more sensitive to Fas-dependent apoptosis and more resistant to TNF-dependent apoptosis. The possible mechanism of this difference is discussed.

**METHODS**. HeLa cells were pretreated with cycloheximide (4 μg/ml) or brefeldin A (2 μg/ml) before TNF (1ng/ml) or anti-Fas AB (dilution 1:6000) were added. The cells were fixed with formaldehyde after 6 hours of TNF or anti-Fas AB treatment. The apoptotic cells were analyzed with DAPI staining. For FACS analysis the cells were treated by Fas and TNFR1 antibodies and FITC labeled secondary antibodies.

**RESULTS.** HeLa cells are very sensitive to TNF and Fas dependent apoptosis. They are dying after 10-12 hours of anti-Fas AB treatment, and after 6-7 hours of TNF/ cycloheximide combined treatment. But the pretreatment of HeLa cells for 3 hours with cycloheximide or brefeldin A (the drug, which destroys Golgi and suppresses protein trafficking and secretion) before the supplement of TNF or anti-Fas AB changes the sensitivity of HeLa cells to Fas- and TNF- dependent apoptosis. The pretreated cells become more sensitive to Fas -dependent apoptosis, but more resistant to TNF-dependent apoptosis (see Fig. 1 and 2 - light spots inside of the apoptotic cells correspond to the condensed chromatin).

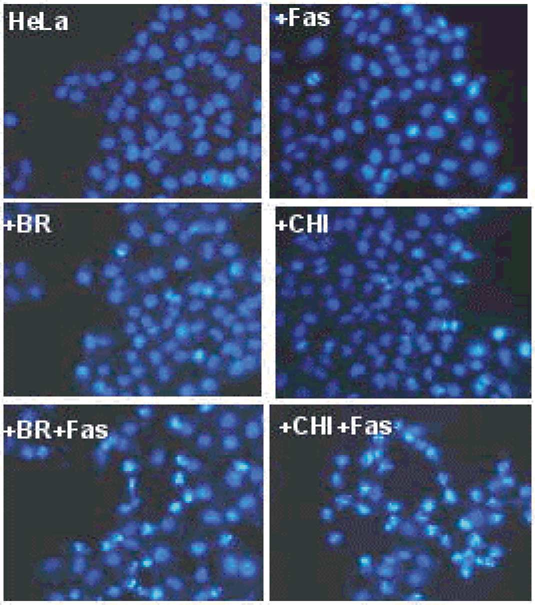

**Fig. 1.** The pretreatment of HeLa cells with brefeldin A or cycloheximide makes them more sensitive to Fas-specific apoptosis.

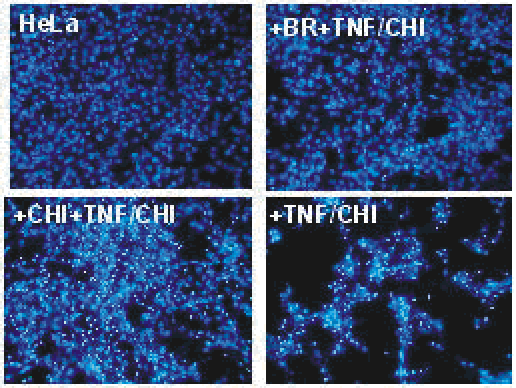

**Fig. 2.** The pretreatment for 3 hours of HeLa cells with brefeldin A (BR) or cycloheximide (CHI) increases their resistance to TNF/CHI.

We analyzed the stability of TNF receptor and Fas on the cell surfaces by FACS to address the mechanism of TNF resistance in brefeldin A and cycloheximide pretreated cells. The results of FACS analysis with AB against TNFR1 and Fas of HeLa cells treated for 3 hours with brefeldin A and cycloheximide are shown at Fig.3. The treatment by brefeldin A and cycloheximide did not change the number of Fas molecules on the cell surface, but decreased the amount of TNFR1 molecules.

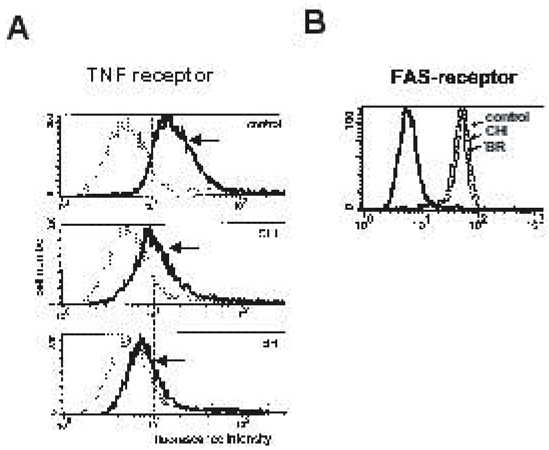

**Fig. 3.** The 3-hours treatment of HeLa cells with brefeldin A or cycloheximide depletes unstable TNFR molecules on cell surface (A), but does not affect presentation of stable Fas on plasma membrane (B).

**DISCUSSION.** According to our results pretreatment of HeLa cells for 3 hours with brefeldin A and cycloheximide has opposite effect on sensitivity of these cells to TNF and Fas dependent apoptosis. The cells become more sensitive to anti-Fas AB treatment, but more resistant to TNF treatment. The mechanism of Fas sensitivity dependence of drug pretreatment is still obscure and can be explained by the degradation of short living proteins with anti-apoptotic activities in the cells. The presence of these proteins was proposed, but still not proved (1). The resistance of the pretreated cells to TNF can be explained by the fact, that TNFR1 in opposite to Fas is very unstable molecule with half-life between 1 and 2 hours (2). For so unstable molecule the suppression of protein synthesis (cycloheximide treatment) or suppression of protein trafficking to the cell surface (brefeldin A treatment) will affect the presentation of this receptor on the cell surface and will make the cells more resistant to TNF-specific apoptosis.

**REFERENCES.**

1. Rokhlin, O.W., Hostager, B.S., Bishop, G.A., Sidorenko, S.P., Glover, R.A., Gudkov, A.V., Cohen, M.B. (1997) Cancer Res. 57, 3941-3943
2. Watanabe, N., Kuriyama, H., Sone, H., Neda, H., Yamauchi, N., Maeda, M., and Niitsu, Y. (1988) J. Biol. Chem. 263, 10262-10266
